# Hidden Infection in Asymptomatic Congenital Lung Malformations—A Decade Retrospective Study

**DOI:** 10.3389/fped.2022.859343

**Published:** 2022-04-14

**Authors:** Chenyu Liu, Xuejiao Yu, Kaisheng Cheng, Dengke Luo, Miao Yuan, Taozhen He, Chang Xu

**Affiliations:** ^1^Department of Pediatric Surgery, West China Hospital of Sichuan University, Chengdu, China; ^2^Department of Clinical Pathology, West China Hospital of Sichuan University, Chengdu, China

**Keywords:** congenital lung malformations, hidden infection, congenital pulmonary airway malformation, bronchopulmonary sequestration, lobectomy, children

## Abstract

**Background:**

Whether to operate on asymptomatic patients with congenital lung malformations (CLMs) remains controversial. Our study intended to find out the proportion of hidden infection in CLMs and its effect on surgery, to provide help for the management of asymptomatic CLMs patients.

**Methods:**

A retrospective review of the medical records of patients with asymptomatic CLMs from January 2011 to December 2020 was performed in our center. Selected asymptomatic patients were divided into a non-hidden infection group (NHI) and a hidden infection group (HI).

**Results:**

A total of 581 asymptomatic CLMs patients were included in this study. Thirty-two percent of asymptomatic CLMs patients had hidden infection in the lesion. Among various CLMs diseases, intralobular pulmonary sequestration had the highest percentage of hidden infection (48.8%). With age, the proportion of HI gradually increased. Patients in the HI and NHI groups were 223 and 121. The incidence of pleural adhesion and focal abscess in the HI group were 14.9 and 7.4%. Statistical significances were shown between the two groups in intraoperative blood loss (*p* = 0.002), operation time (*p* = 0.045), chest tube drainage time (*p* < 0.001), postoperative hospital stay (*p* < 0.001), and air leak (*p* = 0.012).

**Conclusion:**

The proportion of HI detected by postoperative pathological results was high and they could increase the difficulty and risk of surgery. Therefore, early surgery may be a more appropriate choice for the management of asymptomatic CLMs patients.

## Introduction

With the development of ultrasound technology and the frequent use of prenatal screening, the incidence of congenital lung malformations (CLMs) has been increasing in recent years (1, 2). Therefore, pediatric surgeons will encounter more asymptomatic CLMs patients. Over the past few decades, the understanding of the disease has deepened, but controversy still exists (3). The greatest controversy was whether to perform surgical treatment on asymptomatic CLMs patients (4). Surgeons who did not support surgery believed that the trauma caused by surgery was relatively large, the rate of malignant transformation was low, a long-term follow-up would be more beneficial, and surgical intervention should be performed until symptoms appeared (5–7). Doctors who supported surgery believed that early surgery not only had a lower risk of postoperative complications but also had good postoperative lung function compensation in children. Duaa et al. showed that the postoperative exercise capacity of the patients was close to the normal children (8). The researches result from multiple centers have proved that postoperative lung function can be compensated to the normal level (9–11). And most importantly, the surgery eliminates the risk of infection and malignant transformation (12, 13). Such a large controversy makes it difficult for pediatric surgeons to choose strategies for children with asymptomatic CLMs.

Different studies had controversies about the proportion of asymptomatic patients who eventually developed symptoms. Wong et al. showed that 86% of asymptomatic patients eventually developed symptoms, with a median age of two years old (14). However, Thompson et al. found that only 13% of patients eventually developed symptoms through long-term clinical observation (15), which was the core argument over the management of the disease. If the proportion of patients with symptoms is low, conservative management seems to be better; if the proportion of patients with symptoms is high, early surgery may be better, because if the surgery is performed when symptoms occur, the risk and difficulty of surgery, length of hospital stay, and postoperative complications would greatly increase (12, 16). Infection is the most common complication of CLMs, and it is also the focal point for managing asymptomatic CLMs. Past studies have usually focused on children with symptomatic infection but have ignored children with hidden infection. Therefore, the real proportion of infection may be greatly underestimated. For the first time, our study retrospectively analyzed medical records through a large sample size of asymptomatic CLMs patients to provide help for clinical decision making.

## Materials and Methods

This retrospective study was approved by the Ethics Committee of West China Hospital of Sichuan University (file no. 2021-1306). We reviewed the cases of CLMs in our center from January 2011 to December 2020. Patient information and surgery-related information were retrieved from the electronic medical record system. Pathological pictures were obtained from slide scanner systems or reacquired original pathology slides from the pathology slide library. All specimens were routinely stained with hematoxylin and eosin. If the existence of inflammatory cell infiltration was uncertain, immunohistochemistry would be added (LCA, CD3, CD4, CD8, CD15, CD16, or CXCR were used for staining). The pathology pictures were reviewed by two senior pathologists, a third expert was invited when the opinions were inconsistent. The inclusion criteria for hidden infection were patients who had no symptoms related to CLMs since birth but had a pathologically significant number of inflammatory cell infiltration (neutrophils, macrophages, or lymphocytes) under the microscope. Exclusion criteria were CLMs patients who developed symptoms, age >14 years old or missing clinical data. Pathologists and surgeons worked independently, they did not know each other’s results until all the work was done.

Since 2011, multiple surgical methods have been performed in our center, to ensure the consistency of the baseline, asymptomatic patients with CPAM or IPS who underwent thoracoscopic lobectomy were selected to evaluate the impact of hidden infection on surgery. Selected asymptomatic patients were divided into a non-hidden infection group (NHI) and a hidden infection group (HI). Appearances of lesion abscesses and pleural adhesions in the HI group were then recorded through surgical records.

Differences were compared using Pearson’s chi-square test for non-continuous data, Wilcoxon test for continuous non-parametric data, and Student’s *t*-test or Fisher’s exact test for continuous parametric data. *p* < 0.05 was considered statistically significant. SPSS 26.0 statistical software was used for statistical analyses.

## Results

**FIGURE 1:**
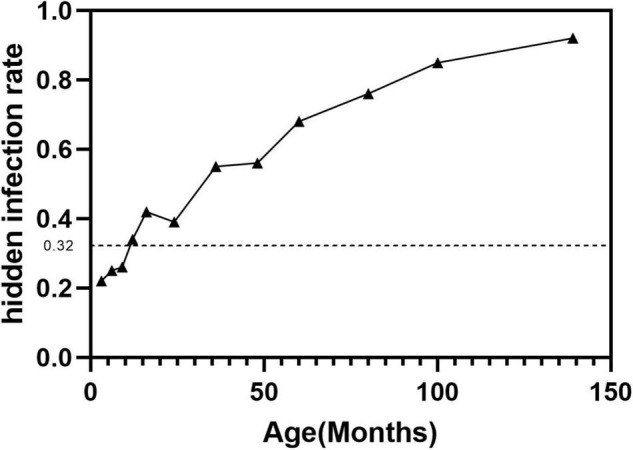
The proportion of hidden infections gradually increased with age.

A total of 581 asymptomatic CLMs patients met the criteria. The characteristics of the study population were shown in Table 1. A total of 186/581 (32%) asymptomatic CLMs patients had hidden infection in the lesion. Hidden infections that can be detected only by H&E staining accounted for 169/186 (90.9%) cases, different immunohistochemical methods were used in 17/186 cases (9.1%). The rate of hidden infection in males was higher than that in females (33.5% vs 29.7%), but the difference was not statistically significant (*p* = 0.338). The characteristics of CPAM and ILS in different lung lobes and stocker classification (17) were shown in Table 2. CLMs lesions located in the left lower lobe or multiple lung lobes had a relatively high proportion of hidden infection. Among CPAM classified by Stocker, the proportion of type 1 CPAM has the highest rate of hidden infection (35.4%). The rates of hidden infection in asymptomatic CLMs patients of different ages were plotted as a line graph (Figure 1). With age, the proportion of hidden infections in the lesions of asymptomatic patients increased.

**TABLE 1:** Characteristics of the study population.

Variables		AP	AH
Gender	Female	232	69 (29.7%)
	Male	349	117 (33.5%)
Basic illness	CPAM	367	103 (28.1%)
	ILS	121	59 (48.8%)
	ELS	67	16 (23.9%)
	BC	14	5 (35.7%)
	CLE	12	3 (25.0%)
Total		581	186 (32.0%)

*AP, aymptomatic CLMs patients; AH, asymptomatic CLMs patients who had hidden infection; CPAM, congenial pulmonary airway malformation; ILS, intralobar pulmonary sequestration; BC, bronchial cyst; ELS, congenital lobar emphysema.*

**TABLE 2:** Characteristics of hidden infection in patients with CPAM or ILS.

Variables	CPAM		ILS	
	AP	AH	AP	AH
*Lobe*				
LUL	49	12 (24.5%)	2	2 (100%)
LLL	101	37 (36.6%)	71	42 (59.2%)
RUL	46	10 (21.7%)	3	1 (33.3%)
RML	14	4 (28.6%)	2	0
RLL	134	29 (21.6%)	33	13 (39.4%)
ML	23	11 (47.8%)	1	1 (100%)
*Stocker type*				
0	0	0		
1	130	46 (35.4%)		
2	186	47 (25.3%)		
3	39	6 (15.4%)		
4	12	4 (33.3%)		
Total	367	103 (28.1%)	121	59 (48.8%)

*LUL, left upper lobe; LLL, left lower lobe; RUL, right upper lobe; RML, right middle lobe; RLL, right lower lobe; ML, multiple lobes.*

Patients in the HI and NHI groups were 223 and 121. Through retrospective analysis of surgical records, intraoperative and postoperative comparisons of the two groups were shown in Table 3. The proportions of pleural adhesions and lesion abscesses in the HI group were 14.9% (18/121) and 7.4% (9/121). The differences in basic illness (*p* = 0.084) and sex (*p* = 0.319) were not statistically significant between the NHI group and the HI group. Five patients were converted to open in the HI group, while none were in the NHI group. There were statistical differences in operation time (*p* = 0.045), blood loss (*p* = 0.002), chest tube duration (*p* < 0.001), and postoperative hospital stay (*p* < 0.001) between the two groups. According to the Clavien–Dindo postoperative complications classification (18), no statistical difference was shown in patients with no complications (*p* = 0.179) between the two groups but were in grade I (*p* = 0.002) and II (*p* < 0.001) complications.

**TABLE 3:** Characteristics of asymptomatic patients with CPAM or ILS who underwent thoracoscopic lobectomy.

Variables		NHI (*n* = 223)	HI (*n* = 121)	*p*
Basic illness	CPAM	167	80	0.084
	ILS	56	41	
Age (month)		8 (6–72)	10 (6–76)	0.048
Sex	Male	141	83	0.319
	Female	82	38	
Conversion to open		0	5	
Operation time (min)		57 (52–76)	61 (55–85)	0.045
Blood loss (ml)		5 (5–15)	10 (5–15)	0.002
Major bleeding		0	0	
Blood transfusions		13	9	0.560
Chest tube duration (day)		2.1 ± 1.2	2.7 ± 1.0	<0.001
Postoperative hospital stay (day)		3 (3–5)	4 (4–5)	<0.001
Air leak		11	15	0.012
Clavien–Dindo classification	None	71 (31.8%)	32 (26.4%)	0.179
	I	117 (52.5%)	43 (35.5%)	0.002
	II	35 (15.7%)	41 (33.9%)	<0.001
	IIIa/b	0	5 (4.1%)	
	IV/V	0	0	

## Discussion

In our study, patients with hidden infection accounted for 32% of all asymptomatic CLMs patients, and the real proportion of infection in CLMs may be greatly underestimated. Compared with previous studies, our study systematically described the proportion of hidden infection on CLMs through a large series and evaluated the impact of HI on surgery. HI would increase the difficulty and risk of surgery and cause more surgical complications, so early surgery may be a better choice for asymptomatic CLMs patients.

**FIGURE 2:**
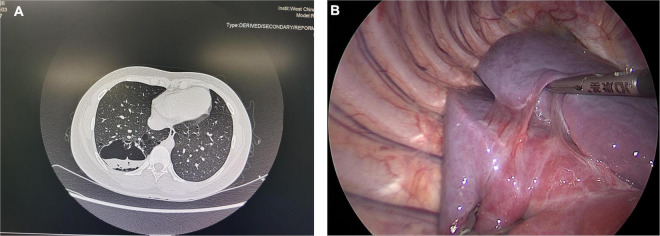
**(A)** A typical CPAM patient with an abscess in the lesion. **(B)** Pleural adhesion in a 7-year-old asymptomatic CPAM patient. These two pictures showed the special situations in asymptomatic CLMs patients.

**FIGURE 3:**
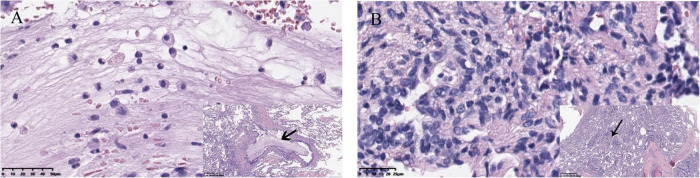
**(A)** Neutrophil infiltration, which always indicates acute inflammation. Viscous exudate in the bronchioles was also found. **(B)** Lymphocyte infiltration, representing chronic infection, and thickening of blood vessel walls could also be seen.

Computed tomography (CT) is currently the most commonly used method for diagnosing CLMs disease after birth, and it has unique advantages compared to ultrasound or MRI (19, 20). In some asymptomatic patients, infection in the lesion could be found through preoperative CT, manifesting as fluid retention in the lesion, which appeared to be lung abscess (Figure 2). However, imaging tests cannot always detect this phenomenon, the pathogens in the lesions were not yet powerful to cause symptoms and imaging changes but could manifest in pathology (Figure 3). Neutrophil infiltration often represents acute infection, and macrophage or lymphocyte infiltration always represents chronic infection. Long-term chronic infection was more common in the HI group. Pelizzo et al. found that 50% (3/6) of asymptomatic CPAM patients had chronic inflammation through histopathologic examination (21). Durelle et al. found that eighteen asymptomatic CLMs patients (26%) had the microscopic disease (22). Microbial evidence has been suggested in asymptomatic CPAM infants for bacteria and fungi (*Pneumocystis jirovecii*, which is often found in immunodeficient patients) (23). Mycobacteria in CLMs lesions have also been reported by several studies (24–26). These opportunistic infections were unlikely to occur in the normal lung, it indicated that there could be immunodeficiency limited in the CLMs lesions, which made the CLMs lesions prone to infection, but the patients did not develop any symptoms. This may be the reason for the high rate of hidden infections in our study, it could also explain the reason of the increasing chance of developing an apparent infection with age.

Different types of CLMs diseases showed different proportions of hidden infections. ILS often appears as lung infection or other CLMs-related symptoms. Zhang et al. showed that up to 71% of ILS patients developed an infection (27). Our study also found that ILS had the highest proportion of hidden infection among various CLMs diseases, while ELS was lower. This was probably because ILs is always connected to normal lung tissue and communicate with the vitro environment, and easier to be invaded by microorganisms. Our study showed that the proportions of asymptomatic infections involving the left lower lobe or multiple lung lobes were higher in children with CPAM. Therefore, when the routine postnatal CT examinations of asymptomatic CPAM patients find that the lesions are located in the above positions, pediatric surgeons should be particularly vigilant. There was an obstruction hypothesis about the etiology of CPAM, which was believed to be the obstruction of the trachea and bronchus in different regions caused the occurrence of CPAM (28). Stocker type 1 CPAM manifests as a cyst diameter greater than 5 mm, so it is also called macrocystic type CPAM (29). Macrocystic CPAM tends to accumulate gas or liquid due to poor drainage and large cavities, which may be the reason why type 1 CPAM is susceptible to hidden infection. A study from Japan showed that the proportion of patients with CLMs-related symptoms gradually increased with age, similar to their research results. Our study found that the proportion of hidden infections also gradually increased with age (30). Older patients were often admitted to the hospital because of symptoms, and the remaining asymptomatic patients had a higher proportion of hidden infections, which indicated that CLMs may have the characteristics of asymptomatic stage and symptomatic stage, patients with hidden infections would gradually develop symptoms as they age.

In our study, patients with hidden infections sometimes presented with pleural adhesions or empyema. Mineo et al. showed that the existence of these two situations may enhance the difficulty and risk of surgery (31). In our study, patients in the HI group had a higher operative time, intraoperative blood loss, and postoperative drainage time and a higher risk of postoperative complications than those in the NHI group. Although no patients had Clavien–Dindo grade IV/V complications, such as organ failure and death, there were statistical differences in grade I–III complications between the two groups, suggesting that more serious complications occurred in the HI group. The hidden infection could lead to larger surgical injury and more exudates, causing longer drainage time and hospital stay, meanwhile, enhancing the risk of air leakage.

Postoperative air leak is the most common complication of thoracic surgery. In our center, the criteria for removing the chest tube are no obvious bubble exudates in the water-sealed bottle when the baby cries, and postoperative CT (X-rays for ELS and BC which the surgery does not involve lung parenchyma) shows good lung recruitment without obvious pneumothorax or pleural effusion. In patients with hidden infections, the surgery was more likely to cause more exudates and leakage in tiny airways or alveoli, resulting in prolonged postoperative drainage time and increasing suffering for the young patients. It is believed that thoracotomy could lead to musculoskeletal deformities (32) and cause acute or chronic pain. Five patients in the HI group experienced conversion to open, while the NHI group did not. Tong et al. found that the most common reason for conversions was vascular injury (33). Pleural adhesion or abscess shielded the pulmonary arteries or veins, increasing the risk of vascular damage during thoracoscopic lobectomy and finally leading to conversions. Therefore, early surgery has a lower risk of hidden infection, reducing the difficulty and risk of surgery, more importantly, eliminating the risk of overt infection and malignant transformation.

With the increasing understanding of CLMs and the maturity of pediatric thoracic surgery, the number of centers supporting surgery on asymptomatic patients has gradually increased in recent years (28, 34). Based on the above reasons, in our center, we also recommend that children with asymptomatic CLMs prenatally diagnosed should undergo thoracoscopic surgery between 6 months and 1 year of age. At this age, children can tolerate the surgery and anesthesia well (35), and the lung can continue to develop to get better compensation (36). Future research should focus on the analysis of the microbial population in the lesion of hidden infection based on next-generation sequencing and immunological research to clarify the mechanism of hidden infection in CLMs patients. Our research was only a single-center retrospective study and lacked monitoring of postoperative lung function. It was unknown whether there was a difference in the long-term prognosis of patients between the NHI and HI groups, but for the first time, our study aimed at the hidden infection of CLMs through a large sample size and analyzed the influence of hidden infection on the operation, which may help pediatric surgeons make clinical decisions about whether to perform an early surgical intervention on asymptomatic CLMs patients.

## Conclusion

We performed a retrospective study on hidden infections of CLMs. The proportion of hidden infections detected by pathology was high, they increased the difficulty and risk of surgery. Therefore, early surgery may be a more appropriate choice for the management of asymptomatic CLMs patients.

## Data Availability Statement

The data for this article are not publicly available according to the hospital and government regulations. Requests to access partial information of these datasets should be directed to CL, lcy_medical@qq.com.

## Ethics Statement

The studies involving human participants were reviewed and approved by Ethics Committee on Biomedical Research, West China Hospital of Sichuan University. Written informed consent to participate in this study was provided by the participants’ legal guardian/next of kin.

## Author Contributions

CL participated in the whole research process. XY provided pathological support. KC, DL, MY, and TH participated in data collection and graph production. CX provided guidance and supervised the conduct of research. All authors read and approved the final manuscript.

## Conflict of Interest

The authors declare that the research was conducted in the absence of any commercial or financial relationships that could be construed as a potential conflict of interest.

## Publisher’s Note

All claims expressed in this article are solely those of the authors and do not necessarily represent those of their affiliated organizations, or those of the publisher, the editors and the reviewers. Any product that may be evaluated in this article, or claim that may be made by its manufacturer, is not guaranteed or endorsed by the publisher.
